# Supplementary material for: Tower Running—Participation, Performance Trends, and Sex Difference
Source: Int J Environ Res Public Health. 2020 Mar 14;17(6):1902. doi: 10.3390/ijerph17061902 (PMC7143174; doi:10.3390/ijerph17061902)
Supplement: Supplementary file 1 [file ijerph-17-01902-s001.pdf]

Supplement 1 R-code:

```
#Packages used
library(readxl)
library(lubridate)
library(plyr)
library(dplyr)
library(stringr)
library(doBy)
library(ggplot2)
library(tableone)
library(gamm4)
library(splines)
library(lme4)
library(lmerTest)
library(mgcv)
library(itsadug)
library(effects)
library(stargazer)
library(ggpubr)
library(readr)

#tower_an=database imported from Excel, recoded to be analysed
#Outcome=speed_km_h (time_m=performance in minutes)
#Effects= Gender, Age, Stairs, Floors
#Year is the year of the Event

#dataset for Figure 1
d3 <- subset(tower_an, !is.na(Gender)) %>%
  group_by(Year, Gender) %>%
  summarise(count=n()) %>%
  mutate(perc=count/sum(count), ratio=count[Gender=="M"]/count[Gender=="F"])

d3<-as.data.frame(d3)

d3_s<-reshape(d3
            , v.names=c("count", "perc")
            , idvar="Year"
            , timevar = "Gender"
            , direction="wide")

d3_s$ratio<-d3_s$count.M/d3_s$count.F
```

```
#Figure 1
ggplot(d3) +
  geom_bar(stat="identity", aes(x=Year, y = count, fill = factor(Gender)), position = "dodge") +
  geom_line(aes(x = Year, y = ratio*1920, group=1, color = 'ratio'), linetype=3, size=1)+
  geom_point(aes(x = Year, y = ratio*1920, group=1, color = 'ratio'), size = 1.5)+
  labs(x = "Year", y = "Climbers (N)", fill = "Sex") +
  scale_y_continuous(expand = c(0,0), breaks=c(0, 500, 1000, 1500, 3000, 5000, 7000), limits=c(0, 7000), sec.axis
= sec_axis(~./1920))+
  scale_x_continuous(breaks=c(2014, 2015, 2016, 2017, 2018, 2019))+
  scale_fill_manual(values = c("pink","cadetblue3")) +
  scale_color_manual('', labels = 'Men to Women ratio', values = 'black') +
  theme_bw(base_size = 18)

#Table 2
#Age/Gender groups: mean(SD)
mean_g<-subset(tower_an, !is.na(Gender) & !is.na(Age)) %>%
        group_by(Age, Gender) %>%
        summarise(count=n(),
              mean=mean(speed_km_h),
              sd=sd(speed_km_h)) %>%
        mutate(ratio=count[Gender=="M"]/count[Gender=="F"])
#t-tests sex groups for each age group
by(tower_an, tower_an$Age, function(.tower_an) t.test(speed_km_h~Gender, data=.tower_an))
#Two-way ANOVA
summary(aov(lm(speed_km_h~Gender+Age, data=tower_an)))

#Table 3 - height in m (tower height in meters)
CreateTableOne(vars=c("speed_km_h", "time_m", "height.in.m", "Floors", "Stairs"), strata = "Gender",
data=tower_an)

#Statistical model: Mixed models: bs=basis splines; Num_name is a number for identify climbers by their names
- this model takes into
#account repeated measurements
fit_g2<-lmer(data=tower_an, speed_km_h ~ Gender*Age*bs(Stairs, 5)+bs(Floors,5)+(1|Num_name))
#Supplemental Table 1
class(fit_g2) <- "lmerMod"

stargazer(fit_g2, type="text", out="rep3.html", star.cutoffs = c(0.05, 0.01, 0.001), keep.stat=c("n"),
      model.numbers=F, no.space=T, column.labels=c("Speed km/h"),
      dep.var.labels.include=F)
```

```
#database for the fitted values
ef <- Effect(c("Gender", "Age", "Stairs"), xlevels=list(Stairs=seq(300, 2500, 50)), fit_g2)
x_22 <- as.data.frame(ef)
ef <- Effect(c("Floors", "Gender"), xlevels=list(Floors=seq(5, 150, 5)), fit_g2)
x_22b <- as.data.frame(ef)

#Figure 2
fig2<-ggplot(subset(tower_an, !is.na(Age)), aes(x=Stairs, y=speed_km_h, color=Gender, linetype=Gender)) +
  #geom_errorbar(aes(ymin=mean-se, ymax=mean+se), width=.1,position=pd) +
  geom_line(data=subset(x_22, fit>0 & fit<7), aes(x=Stairs, y=fit, color=Gender, linetype=Gender), size=1.5) +
  stat_summary(geom = "point", fun.y = mean, size=1)+
  labs(x="Stairs", y="Speed (km/h)", color="Sex")+
  scale_color_manual(values = c("pink","cadetblue3"), labels=c("F", "M")) +
  scale_linetype_manual(name = "Sex"
                      , values=c(1,6)
                      , labels=c("F","M")
  ) +
  facet_wrap(~factor(as.vector(Age), levels = c("<20", "20-29", "30-39", "40-49", "50-59", "60-69", ">69")))+
  # scale_y_continuous(breaks = c(0.08333333, 0.125, 0.1666667, 0.2083333),labels = c("02:00", "03:00", "04:00", "05:00"))+
  theme_bw(base_size = 18)
ggsave("Figure 2.tiff", ggarrange(fig2, legend="bottom"), height=25, width=30, units='cm', compression="lzw", dpi=300)

#Figure 3
fig3<-ggplot(tower_an, aes(x=Floors, y=speed_km_h, color=Gender, linetype=Gender)) +
  #geom_errorbar(aes(ymin=mean-se, ymax=mean+se), width=.1,position=pd) +
  geom_line(data=x_22b, aes(x=Floors, y=fit, color=Gender, linetype=Gender), size=1.5) +
  stat_summary(geom = "point", fun.y = mean)+
  labs(x="Floors", y="Speed (km/h)", color="Sex")+
  # scale_y_continuous(breaks = c(0.08333333, 0.125, 0.1666667, 0.2083333),labels = c("02:00", "03:00", "04:00", "05:00"))+
  scale_color_manual(values = c("pink","cadetblue3"), labels=c("F", "M")) +
  scale_linetype_manual(name = "Sex"
                      , values=c(1,6)
                      , labels=c("F","M")
  ) +
  theme_bw(base_size = 12)
ggsave("Figure 3.tiff", ggarrange(fig3, legend="bottom"), height=12, width=15, units='cm', compression="lzw", dpi=300)
```

Supplemental 2 Table:

Predictor	Speed (km/h)
**Sex** (ref F)	
M	0.881 (0.586)
**Age** (ref 20-29)	
<20	-4.579** (1.519)
30-39	1.765** (0.557)
40-49	2.367*** (0.579)
50-59	6.903*** (0.703)
60-69	1.278* (0.610)
> 69	10.368 (6.616)
**Stairs**	
BS(Stairs, 5)1	1.059 (0.745)
BS(Stairs, 5)2	0.835 (0.491)
BS(Stairs, 5)3	0.859 (0.573)
BS(Stairs, 5)4	0.211 (0.533)
BS(Stairs, 5)5	1.584** (0.559)
**Floors**	
BS(Floors, 5)1	0.033 (0.039)
BS(Floors, 5)2	-0.053 (0.029)
BS(Floors, 5)3	-0.285*** (0.028)
BS(Floors, 5)4	0.587*** (0.046)
BS(Floors, 5)5	0.258*** (0.051)
**SexM:Age**	
SexM:Age< 20	-0.690 (1.893)
SexM:Age30-39	-1.848** (0.607)
SexM:Age40-49	-1.325* (0.624)
SexM:Age50-59	-5.272*** (0.742)
SexM:Age60-69	-0.706 (0.669)
SexM:Age> 69	-14.030* (6.968)
**Sex M:Stairs**	
SexM:*BS*(Floors, 5)1	-0.251 (0.797)
SexM:*BS*(Floors, 5)2	-1.035 (0.528)
SexM:*BS*(Floors, 5)3	-0.526 (0.616)
SexM:*BS*(Floors, 5)4	-0.859 (0.575)
SexM:*BS*(Floors, 5)5	-0.770 (0.600)
**Age:Stairs**	
Age< 20:*BS*(Stairs, 5)1	6.122** (2.085)
Age30-39:*BS*(Stairs, 5)1	-2.287** (0.759)
Age40-49:*BS*(Stairs, 5)1	-3.074*** (0.789)
Age50-59:*BS*(Stairs, 5)1	-9.022*** (0.960)
Age60-69:*BS*(Stairs, 5)1	-1.691* (0.848)
Age> 69:*BS*(Stairs, 5)1	-14.657 (9.294)
Age< 20:*BS*(Stairs, 5)2	3.869** (1.299)
Age30-39:*BS*(Stairs, 5)2	-1.625** (0.501)
Age40-49:*BS*(Stairs, 5)2	-2.259*** (0.521)
Age50-59:*BS*(Stairs, 5)2	-6.426*** (0.625)
Age60-69:*BS*(Stairs, 5)2	-1.587** (0.567)
Age> 69:*BS*(Stairs, 5)2	-9.068 (5.487)
Age< 20:*BS*(Stairs, 5)3	5.273** (1.733)

Age30-39:*BS*(Stairs, 5)3	-1.895** (0.586)
Age40-49:*BS*(Stairs, 5)3	-2.514*** (0.610)
Age50-59:*BS*(Stairs, 5)3	-7.274*** (0.769)
Age60-69:*BS*(Stairs, 5)3	-1.081 (0.712)
Age> 69:*BS*(Stairs, 5)3	-12.012 (7.743)
Age< 20:*BS*(Stairs, 5)4	1.982 (1.626)
Age30-39:*BS*(Stairs, 5)4	-1.652** (0.547)
Age40-49:*BS*(Stairs, 5)4	-2.319*** (0.570)
Age50-59:*BS*(Stairs, 5)4	-6.679*** (0.839)
Age60-69:*BS*(Stairs, 5)4	-2.404 (1.412)
Age> 69:*BS*(Stairs, 5)4	-5.324 (5.660)
Age< 20:*BS*(Stairs, 5)5	12.506* (5.426)
Age30-39:*BS*(Stairs, 5)5	-1.986*** (0.574)
Age40-49:*BS*(Stairs, 5)5	-2.504*** (0.615)
Age50-59:*BS*(Stairs, 5)5	-7.358*** (2.217)
Age60-69:*BS*(Stairs, 5)5	1.751 (4.352)
Age> 69:*BS*(Stairs, 5)5	-25.605 (22.977)
**Sex M:Age:Stairs**	
SexM:Age< 20:*BS*(Stairs, 5)1	0.286 (2.565)
SexM:Age30-39:*BS*(Stairs, 5)1	2.241** (0.828)
SexM:Age40-49:*BS*(Stairs, 5)1	1.384 (0.851)
SexM:Age50-59:*BS*(Stairs, 5)1	6.509*** (1.013)
SexM:Age60-69:*BS*(Stairs, 5)1	0.253 (0.930)
SexM:Age> 69:*BS*(Stairs, 5)1	18.792 (9.748)
SexM:Age< 20:*BS*(Stairs, 5)2	1.200 (1.643)
SexM:Age30-39:*BS*(Stairs, 5)2	1.914*** (0.549)
SexM:Age40-49:*BS*(Stairs, 5)2	1.644** (0.564)
SexM:Age50-59:*BS*(Stairs, 5)2	5.166*** (0.663)
SexM:Age60-69:*BS*(Stairs, 5)2	1.266* (0.624)
SexM:Age> 69:*BS*(Stairs, 5)2	12.446* (5.808)
SexM:Age< 20:*BS*(Stairs, 5)3	0.081 (2.104)
SexM:Age30-39:*BS*(Stairs, 5)3	1.785** (0.639)
SexM:Age40-49:*BS*(Stairs, 5)3	1.161 (0.657)
SexM:Age50-59:*BS*(Stairs, 5)3	5.288*** (0.809)
SexM:Age60-69:*BS*(Stairs, 5)3	0.152 (0.769)
SexM:Age> 69:*BS*(Stairs, 5)3	15.608 (8.133)
SexM:Age< 20:*BS*(Stairs, 5)4	3.225 (1.965)
SexM:Age30-39:*BS*(Stairs, 5)4	1.884** (0.599)
SexM:Age40-49:*BS*(Stairs, 5)4	1.380* (0.617)
SexM:Age50-59:*BS*(Stairs, 5)4	5.208*** (0.873)
SexM:Age60-69:*BS*(Stairs, 5)4	2.068 (1.440)
SexM:Age> 69:*BS*(Stairs, 5)4	7.381 (6.050)
SexM:Age< 20:*BS*(Stairs, 5)5	-6.851 (5.547)
SexM:Age30-39:*BS*(Stairs, 5)5	1.922** (0.630)
SexM:Age40-49:*BS*(Stairs, 5)5	1.463* (0.664)
SexM:Age50-59:*BS*(Stairs, 5)5	5.385* (2.231)
SexM:Age60-69:*BS*(Stairs, 5)5	-2.830 (4.364)
SexM:Age> 69:*BS*(Stairs, 5)5	33.808 (24.159)
Intercept	0.114 (0.543)
Observations	19,851
Note	*p<0.05;**p<0.01;***p<0.001

Table S1. *Regression analysis (mixed model) of speed (km/h) in tower climbing. Estimates and standard errors (SE) of fixed effects are reported. P-values ranges are marked with asterisks (see note). Smoothing terms, basis splines (BS), are denoted with BS(x, 5) t, where x=stairs, floors; t==1,…,5.*
